# Warpage of Injection-Moulded Thin Plates: Numerical Evaluation of Simulation Strategies and Experimental Validation

**DOI:** 10.3390/polym18111310

**Published:** 2026-05-26

**Authors:** Tomaž Kastelic, Nikolaj Mole, Gašper Cafuta, Bojan Starman, Miroslav Halilovič

**Affiliations:** 1 Cafuta d.o.o., Kalce 5j, SI-1370 Logatec, Slovenia; tomaz.kastelic@fs.uni-lj.si (T.K.); gasper.cafuta@cafuta.eu (G.C.); 2 Faculty of Mechanical Engineering, University of Ljubljana, Aškerčeva 6, SI-1000 Ljubljana, Slovenia; nikolaj.mole@fs.uni-lj.si (N.M.); bojan.starman@fs.uni-lj.si (B.S.)

**Keywords:** warpage, injection moulding, thermomechanical simulation, residual stresses, modelling assumptions, Moldex3D, Abaqus

## Abstract

This study presents an experimental and numerical investigation of warpage in injection-moulded ABS plates, with emphasis on the influence of modelling assumptions and residual stress development on warpage prediction. Two sets of processing conditions with different mould-temperature balances were investigated experimentally, and warpage was measured using a coordinate measuring machine (CMM). Filling and packing were simulated using Moldex3D, while warpage was predicted using two integrated Moldex3D solvers and a coupled Moldex3D–Abaqus thermomechanical approach. Although identical thermal input data were used, the three approaches produced noticeably different warpage predictions. The Moldex3D enhanced solver consistently over-predicted warpage magnitude, while the Moldex3D nonlinear solver captured the nonlinear effects but showed unrealistic localised deformation. The thermomechanical approach predicted the warpage shape more accurately for both parameter sets and showed the closest overall agreement with the experimental results.

## 1. Introduction

Although injection moulding has been one of the most widely used manufacturing processes for polymer parts for several decades, accurate prediction of the final part geometry remains challenging. While the filling and packing stages can be simulated with relatively high accuracy [1,2,3], the deformation of the part during cooling and after ejection is still difficult to predict [4,5]. This deformation, commonly referred to as warpage, leads to deviations from the intended geometry and is generally unavoidable in injection-moulded parts. Warpage represents a major source of dimensional inaccuracy, assembly difficulties, aesthetic defects and reduced functional performance. Due to the limited accuracy of warpage prediction, process optimisation often relies on time-consuming trial-and-error approaches.

### 1.1. Mechanisms Inducing Warpage

In essence, warpage arises from the combined effects of (i) non-uniform spatial and temporal temperature and pressure distributions, (ii) material anisotropy and (iii) the mechanical constraint history [6,7].

Non-uniform temperature and pressure distribution are frequently described in the literature using terms such as differential cooling, temperature variance, mould temperature difference [8], unbalanced flow, and overpacking. Differential cooling typically arises from temperature imbalances between opposing mould faces (such as the core and cavity). The side of the part in contact with the hotter mould surface shrinks more after ejection, causing the component to warp toward the hotter side [6,7,9,10]. Pressure variations, primarily driven by unbalanced flow and overpacking, lead to higher density and lower shrinkage near the gate compared with remote regions, resulting in non-uniform regional (area) shrinkage [7,9].

Material anisotropy is a direct result of molecular and fibre orientation that occurs during the filling and packing stages. Because oriented molecules and fibres contract more in the direction of flow than across it, this anisotropic shrinkage again results in warpage [6,7,9]. In addition, the mechanical constraint history plays a critical role. While the part is inside the mould, its shrinkage is physically restricted by the cavity walls. This constraint leads to the development of residual stresses, which are partially released only after ejection, when the part is no longer constrained by the tool [6,7,9,11].

### 1.2. Residual Stresses in Injection Moulded Parts

All of these phenomena manifest as residual stresses in the part, which are partly released through deformation after ejection. Residual stresses are typically categorised into thermally induced and flow-induced residual stress [6,12,13,14,15,16]. Flow-induced residual stresses originate from viscoelastic flow and molecular orientation during filling and post-filling stages and are frozen in by rapid cooling, which prevents complete stress relaxation. These stresses are generally reported to be several orders of magnitude smaller than thermally induced stresses and are, therefore, often neglected in simplified structural analyses [6,13,16,17,18]. Thermally induced residual stresses arise primarily from non-uniform cooling rates and differential thermal contraction as different material layers pass through the glass transition temperature at different times [6,13,15,17,19]. Some authors further distinguish pressure-induced stresses as a separate category, emphasising that the pressure history during packing fundamentally influences the final residual stress state [12,19,20,21].

The residual stress distribution through the thickness of an injection-moulded part is typically characterised by a three-zone profile consisting of tensile stresses at the surface, compressive stresses in the sub-surface layer, and tensile stresses in the core [11,12,13,14,19,21,22]. Surface tensile stresses develop because the initially solidified skin is restrained by mould adhesion and high packing pressure, effectively stretching it while the core remains molten. The compressive sub-surface layer forms as these regions solidify under high pressure, leading to locally increased density. When the external pressure is eventually removed, these layers exert compressive stresses on the surrounding material. Finally, tensile stresses in the core arise because the central region solidifies last at lower pressure, and its thermal contraction is constrained by the already rigid outer layers [6,12,20,21,23,24].

### 1.3. Warpage Prediction

Early developments in warpage prediction were driven by limited computational resources, leading to significant simplifications, such as linear thermoelastic material models [25,26,27]. Initial approaches treated warpage as a direct consequence of non-uniform thermal shrinkage caused by unbalanced cooling [25] or by incorporating an equation of state (pVT) [28]. Jansen and Titomanlio [29] also used a linear elastic model for the solid polymer while assuming that the melt cannot withstand tensile stress.

Subsequent advances introduced viscoelastic material behaviour to account for the time- and temperature-dependent response of polymers. Liu [30] proposed a viscoelastic phase transformation model that captures stress evolution during cooling by representing the solidified polymer using a standard linear solid model and the melt as a viscous fluid. Subsequent studies by Kabanemi and Crochet [31] and Zheng et al. [21] employed fully three-dimensional thermo-viscoelastic formulations to predict residual stresses and warpage. Choi and Im [23] incorporated viscoelastic behaviour during filling and packing but simplified the material response to linear elasticity after de-moulding, arguing that relaxation times become prohibitively long at the low temperatures prevailing at ejection.

A major limitation of viscoelastic modelling approaches is the availability and reliability of detailed material data. To address this, Fan et al. [32] proposed an anisotropic thermo-viscous-elastic residual stress model that reduces material characterisation requirements while maintaining reasonable prediction accuracy. More recently, Turk and Svenšek [33] developed a comprehensive thermo-elastomechanical approach in which the solidified polymer is treated as elastic while the molten core is represented either as a liquid or as a rubbery solid with a strongly reduced shear modulus. By solving the mechanical problem incrementally over time rather than as a single end-of-process step, their method captures the continuous evolution of deformation during cooling.

Contemporary commercial injection moulding simulation packages, such as Autodesk Moldflow and Moldex3D, incorporate many of the above developments in a form suitable for industrial application. Warpage prediction is typically performed using integrated solvers that combine temperature histories obtained from filling and packing simulations with simplified mechanical analyses of the cooling and post-ejection phases. These solvers often include temperature-dependent elastic properties, empirical representations of stress relaxation, and the so-called in-mould constraint effect to account for restricted shrinkage prior to ejection. To improve robustness and computational efficiency, material behaviour is commonly linearised, geometric nonlinearity is treated in a limited or approximate manner, and residual stress evolution is simplified. While such approaches enable efficient warpage prediction for a wide range of practical applications, their simplifying assumptions can limit accuracy when large deformations, strong through-thickness temperature gradients, or pronounced time-dependent relaxation effects dominate the warpage response.

Recent studies have demonstrated that physically enriched finite element frameworks can substantially improve the quantitative accuracy of warpage predictions when detailed material behaviour is accounted for. Alms et al. [34] employed Abaqus in combination with filling and microstructure simulations to predict warpage of semi-crystalline polymers, showing that incorporating through-thickness variations of Young’s modulus is essential for capturing both the correct deformation direction and magnitude. Sun et al. [35,36] developed a two-step simulation workflow integrating modified pVT data and a strain-dependent Young’s modulus within a viscoplastic constitutive framework, achieving warpage prediction accuracy within 2% of experimental measurements. A coherent line of research by the same group has focused on capturing the full transient evolution of deformation during packing, cooling, and ejection using a unified thermo-mechanical modelling framework. Divekar et al. [4,37] employed a temperature-dependent nonlinear material model with stress relaxation to simulate warpage continuously from the packing phase through demoulding, demonstrating improved agreement with experimental measurements compared with conventional end-of-process approaches.

In contrast, several studies evaluating integrated commercial solvers have reported mixed predictive performance. While qualitative trends and parameter sensitivities are often captured correctly [32,38], quantitative agreement with experimental measurements remains inconsistent and strongly dependent on solver formulation, material modelling fidelity, and parameter calibration [39,40,41,42,43,44]. Moreover, most existing studies focus primarily on final deformation metrics, whereas the evolution and spatial distribution of residual stresses, as the fundamental drivers of warpage, are rarely analysed in detail. This lack of mechanistic insight complicates the interpretation of solver discrepancies and limits the ability to identify the physical origins of warpage.

### 1.4. Motivation

In this work, an experimental–numerical framework is presented to investigate warpage of injection-moulded amorphous polymer parts, with particular emphasis on the evolution of residual stresses and their role in deformation. Warpage predictions obtained using two integrated commercial solvers are systematically compared with results from a coupled thermomechanical finite element (FE) approach and validated against detailed experimental measurements. By explicitly linking solver assumptions to residual stress development and resulting warpage, the study aims to clarify the physical mechanisms governing warpage prediction accuracy.

## 2. Materials and Methods

To investigate the mechanisms governing warpage and to evaluate the predictive capabilities of different modelling approaches, an experimental–numerical framework was developed for injection-moulded amorphous polymer parts (ABS).

An experimental campaign was conducted using two distinct sets of processing parameters, during which temperature and pressure were monitored. The final geometry of the cooled parts was measured to quantify warpage.

Filling and packing simulations were performed using Moldex3D. Warpage was subsequently predicted using two integrated approaches available within Moldex3D, as well as an additional thermomechanical approach implemented in Abaqus. The predicted warpage shapes were then qualitatively and quantitatively compared with the experimental results.

### 2.1. Experimental Setup

#### 2.1.1. Part and Mould

**Figure 1:**
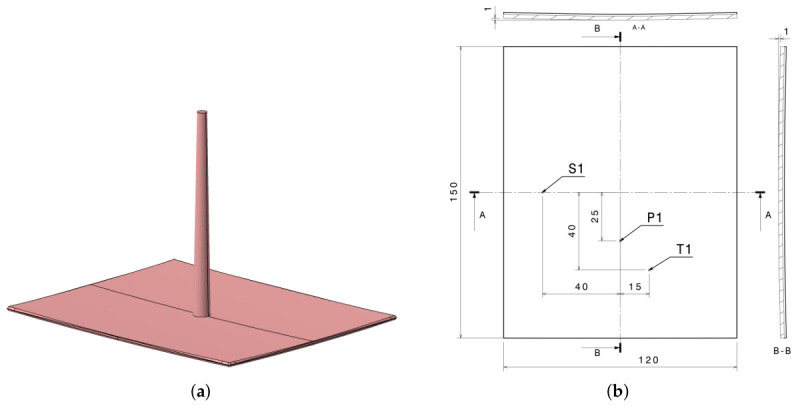
The part used for the study: (**a**) 3D model of the part and runner (**b**) part drawing with dimensions and locations of the pressure sensor (P1), thermocouple (T1), and residual-stress extraction point (S1).

The thin-walled plate geometry previously employed in our earlier study [45] was selected as the test case and is shown in Figure 1. The part consists of a curved plate with dimensions of 150 × 100 × 2 mm. This simple geometry facilitates clear interpretation of the simulation results, while the predefined curvature introduces a well-defined warpage direction, making the part suitable for evaluating differences between experimental and simulated results.

**Figure 2:**
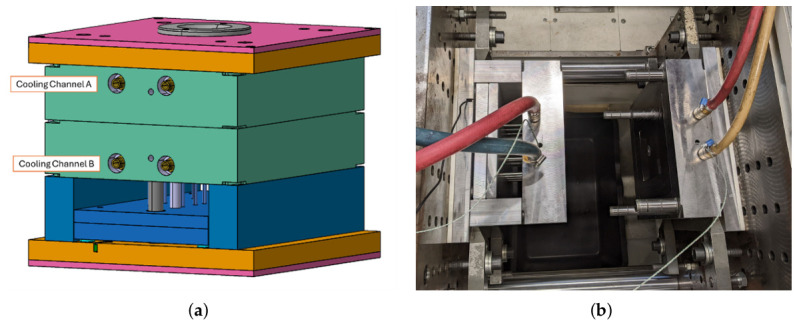
The mould tool used in the study: (**a**) 3D model of the mould (**b**) tool on the injection moulding machine.

The experiment used a mould tool with a runner system, cooling channels, ejectors and two sensors to monitor the injection process (Figure 2). The runner is positioned in the centre of the plate with a diameter of 8.4 mm at the gate. Two cooling channels with a diameter of 10 mm, which can effectively control the mould temperature from both sides, are placed 20 mm from the cavity.

#### 2.1.2. Sensors and Measurement

A temperature sensor and a pressure sensor were installed, and the measured values were later used for validation of the filling and packing simulation.

The pressure sensor was positioned 25 mm from the centre of the part (point P1 in Figure 1b), which corresponds to approximately one third of the flow length from the runner [46]. The sensor is an indirect type and measures pressure through the force applied by the melt on the surface of an ejector pin. The pressure is calculated from the measured force and the pin surface area. Force was measured by a DYHW-116 mini load cell (CALT Sensor, Shanghai Qiyi Co., Ltd., Shanghai, China) rated for 2000 N. The absolute accuracy of the load cell is ±4 N. The load cell was calibrated prior to the experiments on a tensile test bench with a 10 kN load cell (type µTC4, AEP transducers, Cognento, Modena, Italy).

Temperature was measured by a K-type Class 1 thermocouple placed in a drilled hole inside the mould plate (point T1 in Figure 1b), 4 mm from the cavity surface on the cavity side. The thermocouple was electrically insulated using a thermal pad with thermal conductivity of 12 W/(m K). Positioning the sensor close to the melt allows the transient temperature increase and decrease to be captured during each cycle. Sensor accuracy is rated at ±1.5 °C.

The sensors were connected to a DEWE-43A (Dewesoft d.o.o., Trbovlje, Slovenia) data acquisition system, and the measured data were processed in the DewesoftX software (version 2023.4) application. The sampling frequency was set to 20 kHz.

#### 2.1.3. Material

For the case study, an amorphous polymer was selected. The investigated material was ABS (CHI-MEI Polylac PA757), for which material properties are available in the Moldex3D library. In this study, the provided material properties were used without modification in the subsequent simulations. Material models and properties are presented in Appendix A.

#### 2.1.4. Process Parameters

The injection moulding experiments were performed on a KraussMaffei PX 121-540 machine (KraussMaffei Group GmbH, Parsdorf, Germany). The tool was tempered using a water-based temperature control unit Wittman Tempro Primus C 90 (WITTMANN Technology GmbH, Vienna, Austria).

Two sets of process parameters were used and are presented in Table 1. The difference between the two sets lies in the coolant temperature on the core side. In parameter set A, the cavity and core plates were tempered with different coolant temperatures (50 °C and 30 °C), resulting in a through-thickness temperature gradient. Such thermal imbalance typically results in larger warpage [10]. In contrast, in parameter set B, both mould halves were tempered equally (50 °C), leading to more uniform cooling, and the warpage magnitude is expected to be lower.

The experimental scope was limited to two process-parameter sets, which were selected to represent distinct thermal boundary conditions and to enable direct validation of the numerical predictions. Although additional variations in process parameters would be valuable, such cases were outside the available experimental dataset and are, therefore, left for future investigation.

Injection moulding was performed for each parameter set. After the thermocouple temperature stabilised, five consecutive parts were selected for warpage measurement and allowed to cool to room temperature on a table.

#### 2.1.5. Warpage Measurement

**Figure 3:**
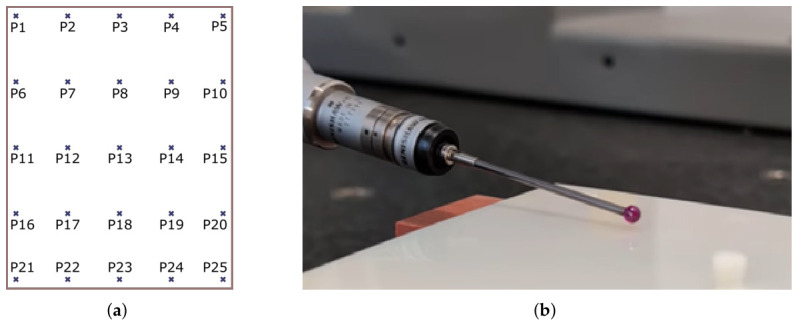
Warpage was measured using a CMM. (**a**) Measurements were performed on a 5 × 5 grid of measuring points. (**b**) CMM probe was positioned at 82.5 ° angle.

The parts were left for two days to reach ambient conditions and stabilise their shape [47]. After this period, a coordinate measuring machine (CMM) was used to measure warpage. Measurements were performed on a Mistral 775 using a spherical probe with a diameter of 4 mm. According to the manufacturer, the accuracy of this configuration is up to ±0.01 mm. The measured part was placed horizontally (runner oriented downwards) and roughly positioned in the nominal coordinate system by probing all four edges and three points on the top surface. Figure 3a shows the measurement grid of 25 distributed probing points used to quantify local deviations from the nominal surface.

To prevent deformation caused by probe contact, the probe was positioned at an angle of 82.5 ° (Figure 3b), which minimised the contact force while maintaining adequate clearance for the measuring head to move over the part.

The measurement results are presented in Section 3.1.2.

### 2.2. Simulation Setup

The numerical warpage prediction is divided into two stages:

1. the injection moulding simulation, including the filling and packing phases, and
2. the warpage simulation, accounting for part deformation during cooling and after ejection.

The injection moulding simulation, covering filling and packing, was performed using the commercial software Moldex3D (version 2024, CoreTech System Co., Ltd., Hsinchu, Taiwan). Warpage was subsequently predicted using three different approaches. Two of these approaches are fully integrated within Moldex3D and were applied using their standard solver formulations. The third approach is an externally coupled workflow, in which temperature data obtained from Moldex3D are transferred to Abaqus/Standard (version 2019, Dassault Systèmes, Vélizy-Villacoublay, France), where the cooling and deformation of the part are simulated using a thermomechanical FE analysis.

The three approaches differ primarily in how the thermal history, material response, geometric nonlinearity, and constraint conditions are treated during the warpage computation. By applying all three approaches to the same part geometry, material model, and processing conditions, their predictive capabilities can be compared under identical thermal input data.

#### 2.2.1. Geometry and FE Mesh

**Figure 4:**
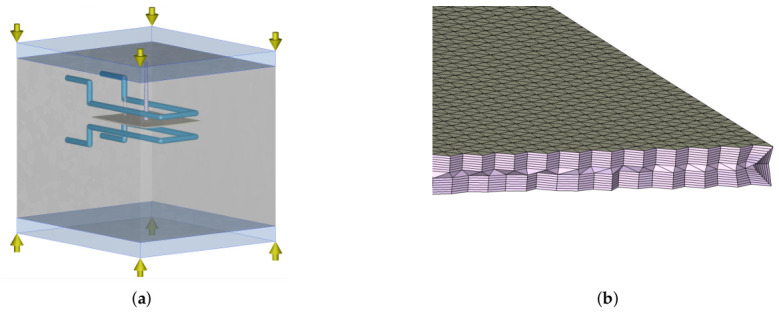
(**a**) 3D geometric model of the mould and (**b**) part meshed with BLM.

For the injection moulding simulation, the geometrical model consisted of the part, runner system and mould base with two cooling channels (Figure 4a).

The part was meshed using a boundary layer FE mesh (BLM) composed of triangular prism FEs in the boundary layers and tetrahedral FEs in the core region. Seven boundary layers were used, and the global FE size for the part was set to 1 mm. FE mesh convergence for this configuration had been verified in a previous study [45], and the same meshing strategy was, therefore, adopted here to ensure consistency. Additional FE mesh metrics are summarised in Table 2.

**Figure 5:**
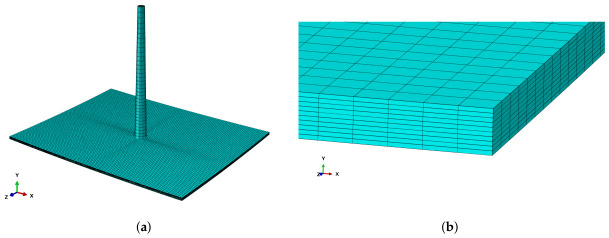
FE mesh adopted for the thermomechanical approach. (**a**) Part and runner are meshed with brick FEs. (**b**) The mesh contained 10 FEs through the thickness.

For the warpage simulation using the thermomechanical approach in Abaqus/Standard, a different geometrical model and mesh were adopted (Figure 5). In this model, the mould was represented by two rigid surfaces, meshed with four-node rigid shell elements (R3D4). The part and runner were merged into a single deformable body and meshed using eight-node brick FEs (C3D8). Ten FEs were used through the part thickness, and the global FE size was set to 1.5 mm, resulting in a total of 85,652 FEs. A mesh-sensitivity study was performed, and the results are presented in Section 3.3.2.

#### 2.2.2. Material Model

The same material model was used consistently throughout all simulation stages. All material behaviour definitions and associated material properties are provided in Appendix A.

#### 2.2.3. Process Parameters

The process parameters applied in the simulations were taken directly from the experimental conditions listed in Table 1. No subsequent parameter adjustment or back-correction was performed based on the measured pressure or temperature histories.

#### 2.2.4. Warpage Simulation

Three approaches were used to calculate the final warpage of the part:

1. the enhanced solver integrated in Moldex3D,
2. the nonlinear warp solver integrated in Moldex3D, and
3. a thermomechanical approach based on coupling Moldex3D and Abaqus/Standard.

The integrated solvers were applied as provided within Moldex3D, without modification of their underlying formulations. The thermomechanical approach was developed to allow explicit control over boundary conditions, contact interactions, and constitutive behaviour during cooling and ejection. A summary of the main characteristics of the three approaches is given in Table 3.

##### Integrated Moldex3D Approaches

The following descriptions of the integrated Moldex3D solvers are based on the available manufacturer documentation [48]. Since the detailed numerical implementation of these proprietary solvers is not fully disclosed, the description is limited to documented solver features and to the authors’ interpretation of the documented workflow.

The enhanced solver uses the transient temperature history obtained from the filling and packing simulation to update temperature-dependent material properties during the warpage calculation. At each time increment, the solver computes the warpage by considering the temperature gradients and the coefficient of thermal expansion. Stress relaxation is then analysed. In the initial stage of the warpage phase, the part is still inside the mould and cannot deform freely, which is considered by the in-mould constraint effect (IMC). After ejection, the warpage calculation proceeds until the part reaches room temperature [48].

The nonlinear warp solver is derived from the standard solver, which calculates warpage using initial strains derived from the PVT response of the material between the end of packing and room temperature within a linear equilibrium formulation. In contrast, the nonlinear warp solver accounts for finite deformation and geometric nonlinearity [48]. The governing equilibrium equation is expressed as

(1) $$\left[K\left(u\right)\right]u=f\left(u\right)$$

where K is displacement-dependent stiffness matrix, u is displacement vector and f is the force vector. This solver is typically applied to thin-walled or shell-like components, for which large deformations and buckling effects are significant. A preliminary linear buckling analysis is used to obtain eigenmodes, which are subsequently introduced as geometric imperfections to initiate nonlinear deformation during the multi-step stress analysis.

##### Thermomechanical Moldex3D-Abaqus Approach

In the thermomechanical approach, Moldex3D was used to compute the transient temperature evolution during filling and packing, and the resulting temperature fields were transferred to Abaqus for the simulation of cooling and deformation. This externally coupled workflow enables explicit definition of contact interactions between the part and the mould surfaces and allows direct control over boundary conditions during cooling and ejection.

The stress analysis was performed using the implicit Abaqus solver with geometric nonlinearity enabled, allowing large-deformation effects to be captured during free shrinkage. Time-dependent viscoelastic stress relaxation and temperature-dependent material properties were included, with both driven by the transient temperature field imported from Moldex3D.

To limit computational cost, a sequentially coupled thermo-mechanical strategy was adopted. First, a heat transfer analysis was carried out to determine the temperature evolution after ejection. The resulting temperature fields were then prescribed as initial and boundary conditions in the subsequent stress analysis.

**Heat Transfer Analysis**

After completion of the injection moulding simulation, transient temperature fields were exported at selected stages of the process and mapped onto the new FE mesh (Figure 5). The exported data span the period from the end of filling through the packing and cooling up to the moment of part ejection.

After ejection, the part continues to cool primarily by convective heat transfer to the surrounding air. To capture this phase, a separate heat transfer analysis was performed. A film boundary condition was applied to all external surfaces of the part, with a convective heat transfer coefficient of 10 W/(m^2^ K), corresponding to typical values for natural convection in air under low-velocity conditions (2–25 W/(m^2^ K)) [49]. The ambient air temperature was set to 23 °C.

**Stress Analysis**

The imported and calculated temperature fields were prescribed as predefined fields for the stress analysis, in which temperature-dependent material properties and viscoelastic behaviour were considered. The stress analysis was divided into several steps, as summarised in Table 4.

In the first step, the part remained in contact with the rigid mould surfaces. Contact was treated as kinematic only and was defined as hard contact in the normal direction and frictional contact in the tangential direction, with a friction coefficient of 0.05. During the ejection step, the mould surfaces were moved apart with a prescribed velocity of 0.2 mm/s.

**Figure 6:**
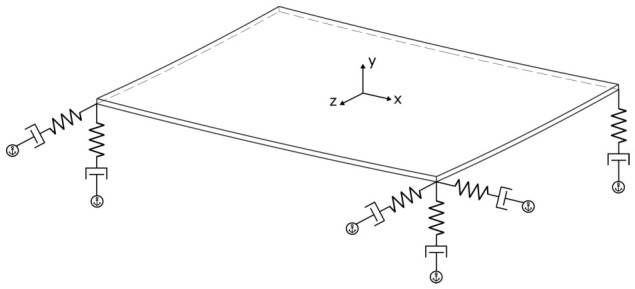
The boundary conditions in Step 3 enable free part deformation and improve simulation stability.

To stabilise the part during subsequent free cooling, six soft connector elements were introduced, connecting three reference points on the part to fixed anchor points in space (Figure 6). The connectors were assigned a low stiffness (k=10N/m) and damping (c=5N·s/m) to prevent rigid body motion while minimising their influence on the mechanical response. This approach provides numerical stability while allowing largely unconstrained deformation of the part.

The warpage calculation was performed using an implicit dynamic analysis, which enables modelling of viscoelastic material behaviour and captures transient effects that may occur during ejection.

**Modelling Assumptions and Limitations**

The thermomechanical approach is subject to several modelling assumptions:

- The coefficient of linear thermal expansion was assumed to be constant over the entire temperature range, consistent with the integrated approaches.
- The elastic response was described using a temperature-dependent Young’s modulus, while linear elasticity was assumed at each temperature level, neglecting potential nonlinear elastic effects.
- The analysis followed a sequentially coupled thermo-mechanical strategy, in which the temperature field was prescribed and mechanical feedback on heat transfer was not considered. As a consequence, possible interactions between deformation-induced contact changes and thermal boundary conditions were not captured.
- Pressure history was not included in this analysis. This choice was made to limit model complexity and because the applied packing pressure of 20 MPa was relatively low for the investigated ABS material, compared with the recommended ABS holding pressure range of 40–90 MPa [50]. Under low-packing-pressure conditions, pressure-induced residual stresses have been reported to be approximately one order of magnitude lower than thermal-induced residual stresses [18]. Therefore, pressure effects were treated as secondary compared with thermal and constraint-driven mechanisms [18,20,24].

The three warpage prediction approaches differ mainly in their treatment of material behaviour, geometric nonlinearity, and constraint conditions during cooling and ejection. The integrated Moldex3D solvers were applied as provided for industrial use, while the thermomechanical approach was used to allow explicit control of boundary conditions and stress evolution.

## 3. Results

### 3.1. Experimental Results

#### 3.1.1. Pressure and Temperature Measurement

The experimental measurements are first presented to verify process stability and to provide a reference for validating the numerical simulation of the filling and packing stages.

**Figure 7:**
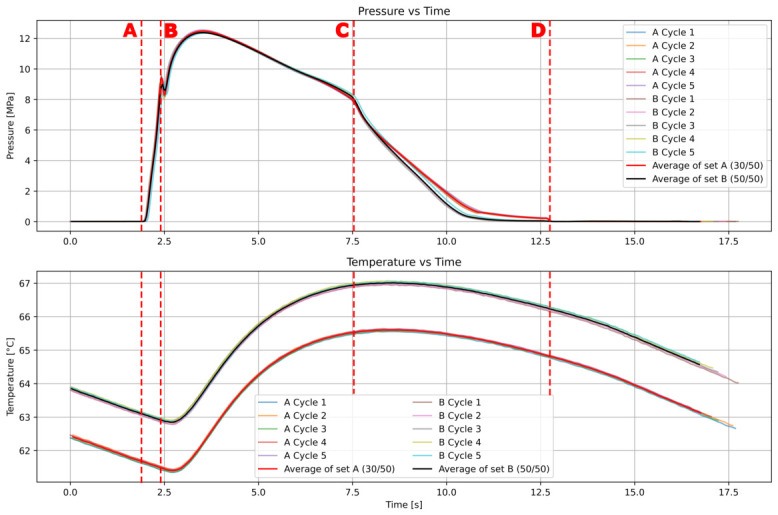
Measured pressure and temperature evolution for each cycle.

During the injection moulding experiment, cavity pressure and mould temperature were recorded for all cycles. Figure 7 shows the measured pressure and temperature profiles for five consecutive cycles. Individual cycle curves are shown together with their average, demonstrating high repeatability of the injection process.

Table 5 shows the maximum values of temperature and pressure for each cycle and the calculated standard deviation. Both pressure and temperature exhibit negligible cycle-to-cycle variation, indicating stable processing conditions.

The pressure curve clearly shows the four characteristic stages of the injection cycle (Figure 7): Dashed line A marks the start of filling, B and C correspond to the start and end of the packing phase, respectively, and line D indicates mould opening.

Comparison of the pressure and temperature curves reveals that the minimum temperature occurs approximately 1 s after the beginning of the cycle, while the maximum temperature is reached during the cooling phase. Since the thermocouple is positioned 4 mm from the cavity surface within the steel mould, this behaviour is expected and reflects the delay associated with heat transfer from the melt to the tool.

#### 3.1.2. Warpage Measurement

**Figure 8:**
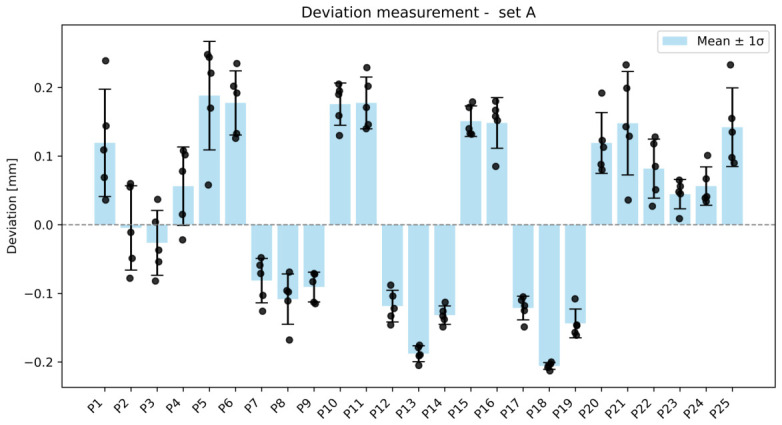
Measured warpage deviations for parameter set A.

**Figure 9:**
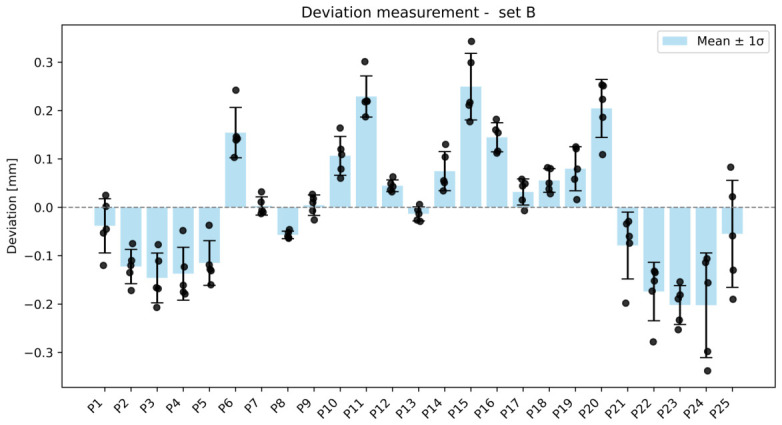
Measured warpage deviations for parameter set B.

For each parameter set, five injected parts were measured. Local deviations from the nominal geometry were quantified at 25 measurement points arranged in a 5 × 5 grid (Figure 3). The measured deviations are shown in Figure 8 and Figure 9 for parameter sets A and B, respectively.

The measurements reveal consistent warpage behaviour within each parameter set, with some scatter between individual samples. This scatter is attributed primarily to unavoidable differences in part handling and positioning during measurement. Nevertheless, the overall deformation modes are clearly distinguishable between the two parameter sets.

**Figure 10:**
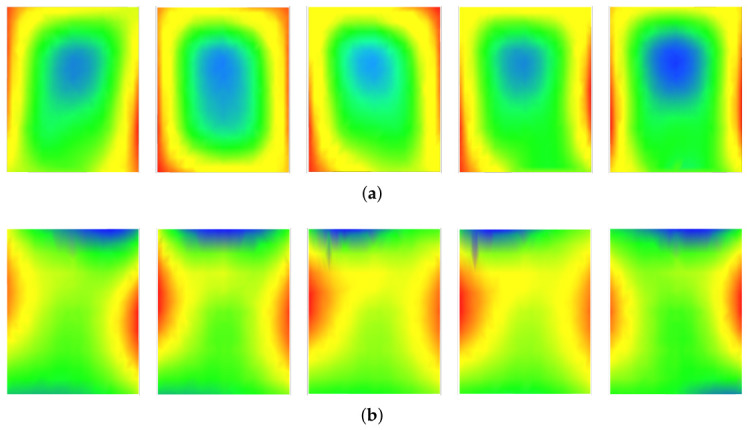
Three-dimensional surface reconstructions were generated from the measured plate specimens and compared with the nominal geometry. The figure provides a qualitative comparison of (**a**) set A and (**b**) set B.

To enable a qualitative comparison of the global deformation mode, the measured deviations were used to reconstruct three-dimensional surface representations of the warped parts. These reconstructed surfaces were compared with the nominal geometry using the open-source software CloudCompare (version 2.12.alpha). The results are shown in Figure 10.

The two parameter sets exhibit distinctly different deformation modes. Parts produced with parameter set A deform into a dome-shaped configuration, while parts produced with parameter set B exhibit a saddle-shaped warpage. These deformation modes correspond to the two typical warpage configurations identified by Shoemaker [7].

The high repeatability of both process and geometry measurements ensures that deviations between simulation and experiment can be attributed primarily to modelling assumptions rather than experimental uncertainties.

### 3.2. Simulation Results

**Figure 11:**
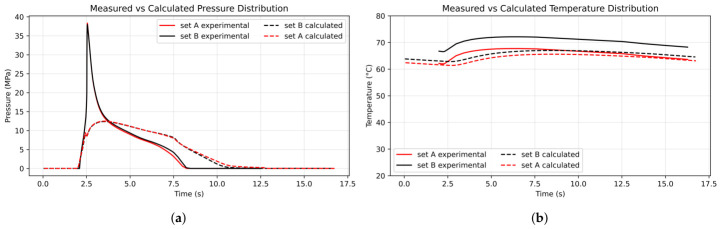
Comparison of measured and simulated time histories for parameter sets A and B: (**a**) pressure at the pressure sensor location P1 and (**b**) temperature at the thermocouple location T1.

All three warpage prediction approaches share the same initial simulation stage, namely the filling and packing analysis performed in Moldex3D. Figure 11a shows the calculated pressure at the location corresponding to the experimental pressure sensor. Figure 11b shows the calculated temperature history at the thermocouple location. For reference, the corresponding experimental measurements are included in both plots.

The agreement between measured and simulated pressure and temperature histories is discussed in Section 3.3.1.

To evaluate the simulated warpage, we focus on the displacement in the global Y-direction, which corresponds to the thickness direction of the part. Out-of-plane displacement is the primary indicator of warpage, while the in-plane components are dominated by uniform shrinkage, and therefore, do not contribute substantially to the warped shape.

**Figure 12:**
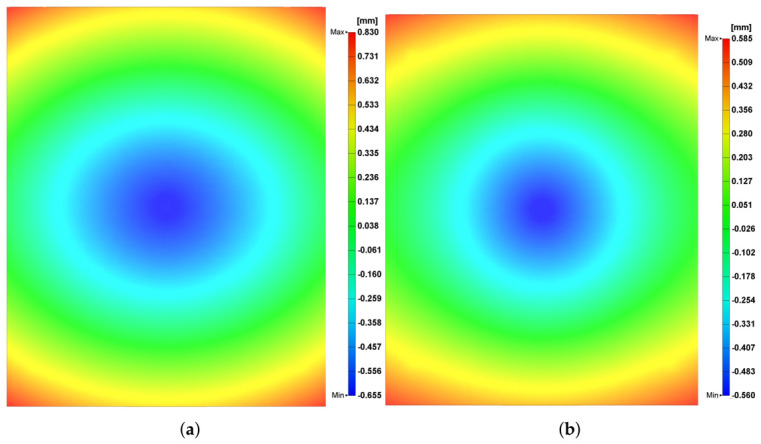
Calculated warpage with enhanced solver for (**a**) set A and (**b**) set B.

**Figure 13:**
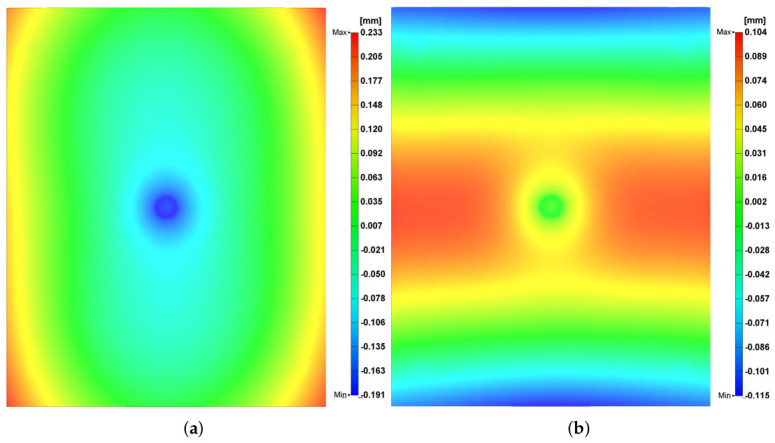
Calculated warpage with nonlinear solver for (**a**) set A and (**b**) set B.

**Figure 14:**
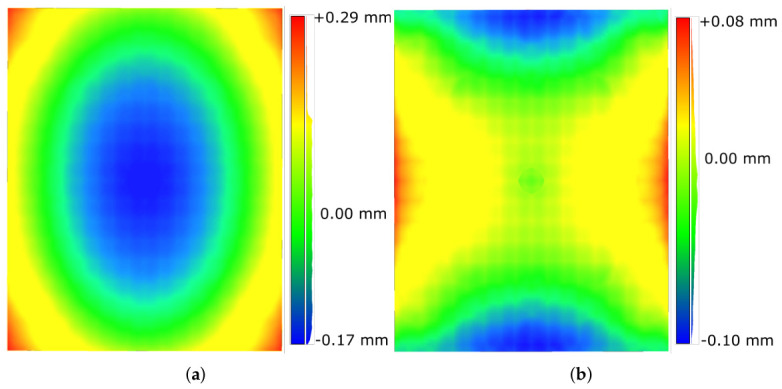
Calculated warpage with the thermomechanical approach for (**a**) set A and (**b**) set B.

Figure 12, Figure 13 and Figure 14 show the calculated Y-direction displacement fields on the top surface of the part for each warpage prediction approach and both parameter sets.

### 3.3. Experiment and Simulation Results Comparison

#### 3.3.1. Process Validation

Figure 11a shows good consistency between the simulated pressure curves for both parameter sets, as well as reasonable agreement with the experimental measurements. Minor discrepancies are observed at the velocity-to-pressure (V/P) switchover point, where the simulation overestimates the pressure. In addition, the simulated pressure decay during packing and cooling is more gradual than in the experiments.

Such discrepancies are commonly reported in injection moulding simulations and are attributed to idealised boundary conditions, including an instantaneous switch to packing pressure, neglect of pressure losses upstream of the cavity, rigid mould assumptions, and the absence of machine and sensor dynamics [51,52,53,54].

The simulated temperature histories exhibit higher absolute values and increased sensitivity to the ejector-side coolant temperature compared with the experimental measurements (Figure 11b). This difference is attributed primarily to the assumption of perfect thermal contact between the mould halves in the simulation, whereas, in the real tool, contact resistance at the parting line reduces heat transfer. Additionally, the thermal inertia of the thermocouple and its electrical insulation contribute to a smoothing of the measured temperature signal.

#### 3.3.2. Abaqus Mesh Sensitivity

To assess the influence of the Abaqus mesh density and the Moldex3D-to-Abaqus data mapping on the thermomechanical warpage prediction, an additional mesh-sensitivity study was performed. The same Moldex3D temperature fields and identical mapping procedure were used for three Abaqus meshes. The results are summarised in Table 6.

The coarse mesh underpredicts the deformation magnitude, indicating that insufficient mesh resolution affects the calculated warpage. In contrast, the baseline and refined meshes give comparable results. Refining the mesh from 85,652 to 233,997 elements changed the RMS deviation from 0.12 mm to 0.11 mm and the deviation range from 0.40 mm to 0.35 mm. The deformation magnitude and interpretation of the results, therefore, remain consistent between the baseline and refined meshes. The baseline mesh was consequently considered sufficiently refined for the thermomechanical analysis.

#### 3.3.3. Warpage Comparison

To enable a quantitative comparison between experimental and simulated warpage, five complementary metrics were defined based on deviations at the 25 measurement points shown in Figure 3a. These include the maximum positive and maximum negative deviations, which characterise extreme local warpage; the deviation range; the average deviation and the root-mean-square (RMS) deviation, which provides a global measure of overall deformation magnitude. Together, these metrics allow assessment of both local extrema and the global deformation state of the part.

For the simulation results, each metric was calculated from the displacement field obtained from a single deterministic simulation for each approach. For the experimental results, the same metrics were first calculated separately for each of the five measured specimens. The mean value, sample standard deviation, and 95% confidence interval of the experimental mean were then determined for each metric. The simulation results were compared with these confidence intervals to assess whether the differences exceeded the experimental scatter.

Furthermore, a root-mean-square error (RMSE) analysis was performed to determine the global discrepancy between the measured and simulated warpage fields. For each specimen, the RMSE was calculated from the differences between the measured and simulated deviations at the 25 corresponding measurement points. The mean RMSE and sample standard deviation were then calculated from the five specimens for each simulation approach. The RMSE and deformation-mode comparison were used as complementary shape descriptors, since scalar metrics alone cannot fully distinguish between different global warpage modes.

In addition, a qualitative comparison of the resulting surface shape is included to assess the overall warpage mode that cannot be fully captured by scalar metrics.

Table 7 and Table 8 summarise the experimental warpage measurements and the corresponding simulation results for parameter sets A and B, respectively.

For parameter set A (Table 7), all three approaches predict the correct overall deformation mode, whereas the thermomechanical approach shows the closest agreement with the experimental measurements. In comparison, the enhanced solver considerably over-predicts the warpage magnitude; its deviation range and RMS deviation are well above the experimental confidence intervals. The nonlinear approach predicts a lower warpage magnitude, with the deviation range and RMS deviation below the experimental confidence intervals, and exhibits highly localised displacements in the negative Y-direction concentrated in a small region around the runner. The quantitative metrics, therefore, show that the thermomechanical approach provides the closest agreement with the experiment. Although some scalar metrics are slightly below the experimental confidence intervals, the absolute differences are small relative to the overall warpage magnitude. Together with the qualitative deformation-mode comparison, the RMSE analysis indicates that the thermomechanical approach provides the best overall agreement with the experimental results.

For parameter set B (Table 8), more pronounced differences between the approaches are observed. While the enhanced and nonlinear solvers fail to predict the experimentally observed saddle-shaped warpage, the thermomechanical approach correctly captures the overall deformation mode. As in parameter set A, the enhanced solver over-predicts the warpage magnitude, with the deviation range and RMS deviation exceeding the experimental confidence intervals. In contrast, the nonlinear and thermomechanical approaches under-predict the deformation magnitude; their deviation ranges and RMS deviations are below the corresponding experimental confidence intervals. Although the quantitative metrics and RMSE values of the nonlinear and thermomechanical approaches are similar, the qualitative deformation-mode comparison shows that the thermomechanical approach provides the better shape-wise agreement with the experimentally observed saddle-shaped warpage. This indicates that the thermomechanical approach captures the dominant deformation mode, but that neither approach fully captures the warpage magnitude under these processing conditions.

#### 3.3.4. Residual Stress Results

To further clarify the origin of the observed differences in warpage predictions, the residual stress distributions obtained from the three simulation approaches were analysed.

**Figure 15:**
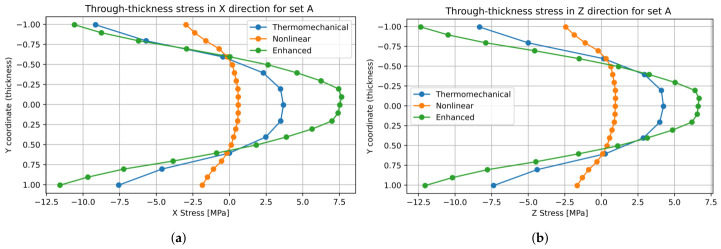
Through-thickness residual stresses at point S1 for parameter set A: (**a**) X direction and (**b**) Z direction.

**Figure 16:**
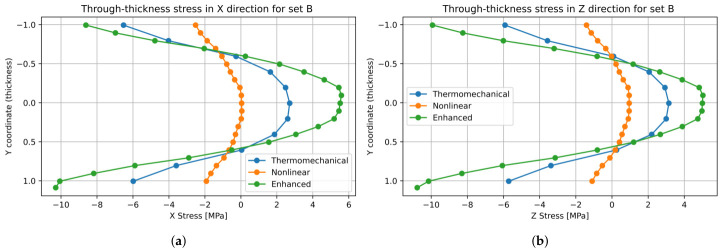
Through-thickness residual stresses at point S1 for parameter set B: (**a**) X direction and (**b**) Z direction.

Table 9 shows the in-plane residual stress distributions in the X direction for both parameter sets. Figure 15 and Figure 16 present the through-thickness residual stress profiles at point S1, located on the YZ symmetry plane and 40 mm from the part centre (Figure 1b).

The residual stress distributions presented in this section are simulation-based results. They are used to support the interpretation of the different warpage predictions, but were not independently confirmed by experimental residual-stress measurements.

## 4. Discussion

The results presented in the previous sections demonstrate that the three investigated simulation approaches lead to noticeably different warpage predictions, despite being based on identical geometry, material data, and initial process simulation input. These differences arise primarily from the underlying modelling assumptions employed in the individual warpage solvers rather than from differences in the thermal boundary conditions.

Across both parameter sets, the thermomechanical approach consistently reproduces the experimentally observed warpage shapes more accurately than the integrated solvers. This indicates that explicit representation of contact conditions, constraint evolution during cooling, and deformation-induced stress redistribution plays an important role in warpage prediction, even when using the same temperature history. However, the comparison also shows that agreement must be evaluated in terms of both deformation mode and magnitude. For parameter set A, the thermomechanical approach reproduced the measured deviation range of 0.40 mm, while the enhanced solver over-predicted it by a factor of approximately 3.3. The nonlinear solver gave a similar deviation range of 0.34 mm, but the corresponding deformation was strongly localised near the runner and was, therefore, not representative of the measured global shape. For parameter set B, the thermomechanical approach captured the experimentally observed saddle-shaped mode, but underestimated the deviation range, predicting 0.15 mm compared with the measured 0.47 mm. In contrast, the enhanced solver again over-predicted the magnitude, while neither integrated solver reproduced the measured deformation mode. These differences suggest that certain simplifications inherent to the integrated approaches are not sufficient to capture the full warpage behaviour for the investigated geometry.

The nonlinear solver shows pronounced stress concentrations, particularly in the runner region, where compressive residual stresses reach values of approximately 40 MPa. These stresses are interpreted as a consequence of material shrinkage combined with the absence of viscoelastic stress relaxation in this approach. This interpretation is supported by an additional sensitivity check, in which removing the structural Maxwell model from the material definition did not change the nonlinear solver result, indicating that viscoelastic relaxation was not active in this approach. As a result, stresses remain largely frozen in the material and are only partially relieved through deformation. This explains the localised stress and displacement field around the runner, while stresses further away from this region decrease to significantly lower levels. The observed behaviour indicates that for the investigated case, geometric nonlinearity alone is insufficient to produce a realistic redistribution of residual stresses during cooling and ejection.

In the enhanced solver, viscoelastic stress relaxation is accounted for, which results in smoother residual stress distributions and the absence of strong stress concentrations near the runner. However, although the detailed numerical implementation of the IMC effect is not disclosed in the available Moldex3D documentation, the residual stress and warpage results suggest that this constraint is represented through a simplified internal formulation. This may contribute to the exaggerated tensile and compressive stresses observed, particularly in the outer regions of the part. Furthermore, unlike the nonlinear solver, the enhanced solver is not documented as resolving finite deformation or geometric nonlinearity. Consequently, stress relaxation through deformation is limited, resulting in an over-prediction of warpage magnitude and, in some cases, incorrect deformation shapes.

Residual stress distributions obtained from the three approaches further clarify these differences. The nonlinear solver produces substantially higher and more localised stresses, while the enhanced solver yields smoother but exaggerated through-thickness stress profiles. The thermomechanical approach results in more uniformly distributed residual stresses and a consistent two-zone through-thickness stress profile. However, in the context of injection-moulded parts, a three-zone residual-stress profile has been reported in many previous studies [11,12,13,14,19,20,21,22,24,55]. Jansen [20] and Zoetelief [12] stated that tensile stresses at the surface are specifically induced by high mould pressure during the packing stage. This is consistent with the stress distributions calculated by other authors [30,31,34], who did not account for the pressure history and thus calculated the two-zone stress profile. Pressure effects in the integrated Moldex3D solvers are included implicitly through the PVT-based initial strain and shrinkage fields, whereas in the thermomechanical approach, the pressure history was not explicitly transferred as a mechanical load or initial stress field. Despite these different treatments of pressure effects, all three approaches produced a two-zone through-thickness stress profile in the present study. Pressure history was not included in thermomechanical approach for the reasons described in list Modelling Assumptions and Limitations. However, at higher packing pressures, the effect of pressure is expected to be more pronounced and would need to be accounted for explicitly. In such cases, mould compliance should also be taken into account since it greatly influences the pressure history [56,57,58,59,60].

The difference between parameter sets A and B further indicates that the accuracy of the simulation depends on whether the dominant warpage-driving mechanism is explicitly represented. In parameter set A, the mould temperature difference creates a pronounced through-thickness thermal imbalance, leading to asymmetric cooling and shrinkage. This is reflected in the thermomechanical approach, where higher compressive stresses are obtained on the side in contact with the hotter mould surface (Figure 15). From a mechanical point of view, this asymmetric stress profile generates a bending moment after the constraints are released, which is consistent with the experimentally observed warpage direction. In parameter set B, the temperature difference between the mould sides is reduced. As a result, pressure-induced stresses, local constraint conditions, and mould compliance may, therefore, have a relatively larger influence. Since these effects are only partly represented in the present thermomechanical approach, the resulting bending moment may be underestimated, which explains why the saddle-shaped deformation mode is captured while the warpage magnitude remains too low.

Overall, the results show that differences in warpage prediction between the investigated approaches are closely linked to the way residual stresses are generated and relaxed during cooling and ejection. Approaches that neglect viscoelastic stress relaxation or use simplified constraint conditions tend to produce unrealistic stress distributions, leading to inaccurate warpage predictions. The thermomechanical approach demonstrates that improved agreement with experimental results can be achieved by reducing these simplifications, even without introducing additional material parameters.

It should be noted that this study was limited to an unfilled amorphous polymer. Therefore, the conclusions should be interpreted primarily for comparable amorphous thermoplastics. Extending the conclusions to semi-crystalline or fibre-filled materials would require additional validation, since crystallisation kinetics and fibre-orientation-induced anisotropy may significantly affect warpage. In such cases, appropriate crystallisation modelling coupled to the temperature and pressure history, or fibre-orientation modelling with the resulting anisotropic properties, should be included.

## 5. Conclusions

This work presented a systematic experimental and numerical investigation of warpage in injection-moulded ABS plates with particular emphasis on the role of residual stresses and modelling assumptions in warpage prediction. Two distinct processing conditions were investigated, differing in mould temperature balance. Experimental measurements confirmed highly repeatable processing conditions and warpage responses, providing a robust basis for numerical model validation.

A comparison of three warpage prediction approaches revealed substantial differences in warpage, despite being based on the same filling and packing simulation input. The enhanced solver consistently over-predicted warpage magnitude, while the nonlinear solver captured the warpage magnitude but neglected viscoelastic stress relaxation. It also failed to correctly predict the observed deformation mode. The thermomechanical approach achieved the best overall agreement with experimental results by explicitly resolving contact constraints, geometric nonlinearity, and time-dependent material behaviour.

For cases in which a pronounced mould temperature difference is present, the thermomechanical approach was capable of predicting warpage in close agreement with experimental measurements, both in terms of deformation mode and warpage magnitude. In contrast, for the second processing condition, where thermal asymmetry was reduced, additional mechanisms were found to influence warpage behaviour. This is reflected in the numerical results, where the thermomechanical approach accurately captured the deformation mode but underestimated the warpage magnitude.

These findings indicate that modelling assumptions related to pressure history during packing and mould deformation may play a significant role in accurately predicting warpage, particularly when the temperature difference is not the dominant driving mechanism. Incorporating these effects is, therefore, expected to further improve the predictive capability of thermomechanical warpage simulations and should be addressed in future work.

## Figures and Tables

**Table 1:** Two sets of process parameters used in the experiment.

Parameter	Parameter Set A	Parameter Set B
Cavity Side Temperature	50 °C	50 °C
Core Side Temperature	30 °C	50 °C
Melt Temperature	240 °C	
Holding Force	800 kN	
Ram Speed	50 mm/s	
Injection Pressure	100 MPa	
Velocity/Pressure Switch	42 MPa, 75 mm	
Injection Time	0.66 s	
Packing Pressure	20 MPa	
Packing Time	5.00 s	
Cooling Time	5.00 s	
Mould-Open Time	6.50 s	
Ejection Timing After Mould Open	3.20 s	

**Table 2:** Mesh metrics for the injection-moulding simulation.

Component	FE Mesh Type	Seed Size	Number of FEs
Part	BLM	1 mm, 7 layers	544,241
Runner	Prisms	0.8 mm	17,784
Cooling Channels	Prisms	1.5 mm	29,984
Mould base	Tetrahedral	20 mm	451,738

**Table 3:** The three approaches used to calculate part warpage.

No.	Approach	Tool	Comment
1	Enhanced	Integrated in Moldex3D	Accounts for VE and temperature history
2	Nonlinear	Integrated in Moldex3D	Accounts for geometrical nonlinearity
3	Thermomechanical	Moldex3D → Abaqus/Standard	Full thermomechanical approach

**Table 4:** Simulation steps of thermomechanical approach.

Step	Phase	Duration	Boundary Condition
Step 1	Packing and Cooling Phase in Tool	10 s	Part in kinematic contact with the cavity surface
Step 2	Ejection	0.2 s	Cavity surface rigid bodies move apart
Step 3	Cooling outside the tool	1200 s	3-2-1 Soft BC (springs and dashpots)

**Table 5:** Average and standard deviation (SD) of maximum pressure and temperature values of all measured cycles.

	Pressure [MPa]		Temperature [°C]	
	parameter set A	parameter set B	parameter set A	parameter set B
mean	12.48	12.37	65.61	67.01
SD	0.04	0.03	0.03	0.05

**Table 6:** Sensitivity of the thermomechanical warpage prediction to the Abaqus mesh density.

Metric	Coarse Mesh	Baseline Mesh	Refined Mesh
Number of elements	34,896	85,652	233,997
Elements through thickness	7	10	13
Global mesh size	2.0	1.5	1.0
Minimum deviation [mm]	−0.12	−0.21	−0.19
Maximum deviation [mm]	0.10	0.19	0.17
Deviation range [mm]	0.21	0.40	0.35
Mean deviation [mm]	0.00	0.00	0.00
RMS deviation [mm]	0.07	0.12	0.11

**Table 7:** Experimental warpage of set A compared with warpage predictions calculated using all three approaches.

Metric [mm]	Experiment ^1^	Enhanced	Nonlinear	Thermomechanical
max. − deviation	−0.21±0.01	−0.78	−0.20	−0.21
max. + deviation	0.23±0.02	0.54	0.14	0.19
deviation range	0.44±0.02	1.32	0.34	0.40
mean deviation	0.02±0.00	0.00	0.00	0.00
RMS deviation	0.14±0.01	0.39	0.08	0.12
RMSE ^2^	–	0.32±0.02	0.08±0.004	0.07±0.01
deformation mode	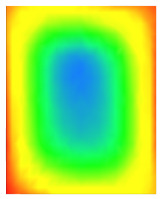	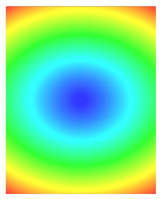	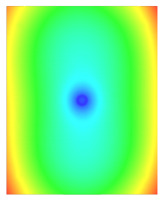	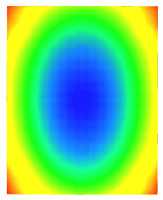

^1^ Experimental scalar metrics are given as mean ± 95% confidence interval of the mean, calculated from five specimens. ^2^ RMSE values are given as mean ± sample standard deviation, calculated from pointwise differences between each measured specimen and the corresponding simulation result.

**Table 8:** Experimental warpage of set B compared with warpage predictions calculated using all three approaches.

Metric [mm]	Experiment ^1^	Enhanced	Nonlinear	Thermomechanical
max. − deviation	−0.25±0.10	−0.39	−0.09	−0.08
max. + deviation	0.27±0.07	0.66	0.10	0.07
deviation range	0.52±0.17	1.05	0.19	0.15
mean deviation	0.00±0.00	0.00	0.00	0.00
RMS deviation	0.14±0.04	0.29	0.07	0.04
RMSE ^2^	–	0.26±0.01	0.09±0.02	0.10±0.03
deformation mode	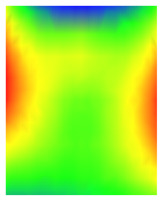	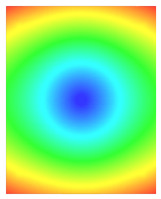	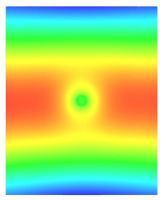	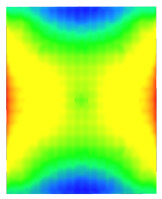

^1^ Experimental scalar metrics are given as mean ± 95% confidence interval of the mean, calculated from five specimens. ^2^ RMSE values are given as mean ± sample standard deviation, calculated from pointwise differences between each measured specimen and the corresponding simulation result.

**Table 9:** Residual stresses in the X direction in the part (MPa) for parameter sets A and B using different solvers.

	Parameter Set A	Parameter Set B
Enhanced	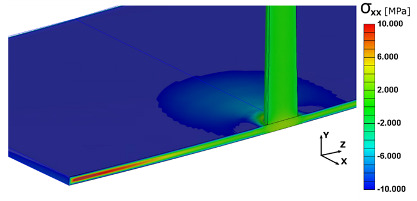	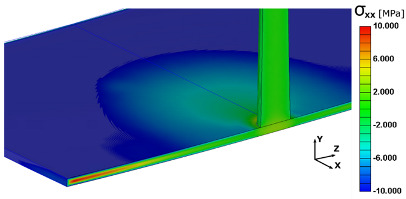
Nonlinear	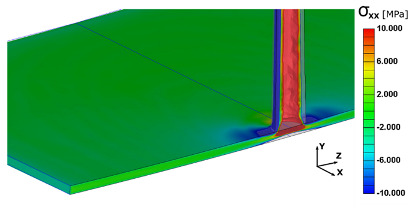	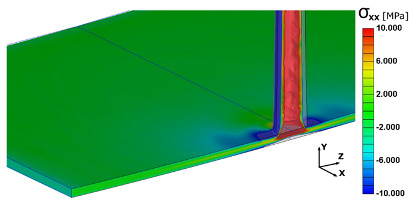
Thermomechanical	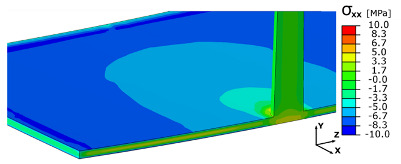	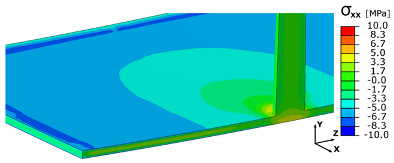

## Data Availability

The raw data supporting the conclusions of this article will be made available by the authors on request.

## Appendix A. Material Data for ABS Polylac PA757

### Appendix A.1. Viscosity: Modified Cross Model

(A1)
$$\eta =\frac{\eta_{0}}{1+{\left(\frac{\eta_{0}\dot{\gamma }}{\tau^{*}}\right)}^{1-n}}$$

(A2)
$$\eta_{0}=D_{1}\exp\left(\frac{-A_{1}\left(T-T_{c}\right)}{A_{2}+\left(T-T_{c}\right)}\right)$$

(A3)
$$T_{c}=D_{2}+D_{3}P$$

(A4)
$$A_{2}={\bar{A}}_{2}+D_{3}P$$

**Table A1:** Parameter values for Modified Cross Model.

Parameter	Value	Unit
*n*	0.21124	-
$\tau^{*}$	1 $\times \;10^{5}$	Pa
$D_{1}$	3.2711 $\times \;10^{15}$	Pa·s
$D_{2}$	363.15	K
$D_{3}$	0	K/Pa
$A_{1}$	37.737	-
${\bar{A}}_{2}$	51.6	K

### Appendix A.2. pvT: Modified Tait Model

(A5)
$$\begin{array}{cc} \hat{V} & ={\hat{V}}_{0}\left[1-C\ln\left(1+\frac{P}{B}\right)\right]+{\hat{V}}_{t} \end{array}$$

(A6)
$$\begin{array}{cc} {\hat{V}}_{0} & =\left\{\begin{array}{cc} b_{1S}+b_{2S}\bar{T}, & \mathrm{if}\;T\leq T_{t}\\ b_{1L}+b_{2L}\bar{T}, & \mathrm{if}\;T>T_{t} \end{array}\right. \end{array}$$

(A7)
$$\begin{array}{cc} B & =\left\{\begin{array}{cc} b_{3S}\exp\left(-b_{4S}\bar{T}\right), & \mathrm{if}\;T\leq T_{t}\\ b_{3L}\exp\left(-b_{4L}\bar{T}\right), & \mathrm{if}\;T>T_{t} \end{array}\right. \end{array}$$

(A8)
$$\begin{array}{cc} {\hat{V}}_{t} & =\left\{\begin{array}{cc} b_{7}\exp\left(b_{8}\bar{T}-b_{9}P\right), & \mathrm{if}\;T\leq T_{t}\\ 0, & \mathrm{if}\;T>T_{t} \end{array}\right. \end{array}$$

(A9)
$$\begin{array}{cc} \bar{T} & =T-b_{5},\;T_{t}=b_{5}+b_{6}P \end{array}$$

**Table A2:** Parameter values for Modified Tait Model.

Parameter	Value	Unit
*C*	8.94 $\times \;10^{-2}$	-
$b_{1L}$	9.7578 $\times \;10^{-4}$	m^3^/kg
$b_{2L}$	4.8067 $\times \;10^{-7}$	m^3^/kg·K
$b_{3L}$	1.6916 $\times \;10^{8}$	Pa
$b_{4L}$	0.003364	1/K
$b_{1S}$	9.7587 $\times \;10^{-4}$	m^3^/kg
$b_{2S}$	1.4467 $\times \;10^{-7}$	m^3^/kg·K
$b_{3S}$	2.153 $\times \;10^{8}$	Pa
$b_{4S}$	1.922 $\times \;10^{-3}$	1/K
$b_{5}$	361.15	K
$b_{6}$	2.2222 $\times \;10^{-7}$	K/Pa
$b_{7}$	0	m^3^/kg
$b_{8}$	0	1/K
$b_{9}$	0	1/Pa

### Appendix A.3. Specific Heat

**Table A3:** Specific heat dependent on temperature.

No.	Specific Heat [J/kg·K]	Temperature [K]
1	1391	305.15
2	1490	323.15
3	1636	353.15
4	1885	371.15
5	2085	377.15
6	2115	383.15
7	2171	403.15
8	2260	433.15
9	2331	463.15
10	2364	483.15
11	2375	493.15
12	2388	513.15

### Appendix A.4. Thermal Conductivity

**Table A4:** Thermal conductivity dependent on temperature.

No.	Thermal Conductivity [W/m·K]	Temperature [K]
1	0.200	333.15
2	0.209	363.15
3	0.217	393.15
4	0.224	423.15
5	0.229	453.15
6	0.235	483.15
7	0.237	513.15

### Appendix A.5. Viscoelasticity: White-Metzner Model

(A10) $$\begin{array}{cc} \tau +\lambda \;\overset{\nabla }{\tau } & =\eta \left(T,P,\dot{\gamma }\right)\left(\nabla v+\nabla v^{T}\right) \end{array}$$

(A11) $$\begin{array}{cc} \overset{\nabla }{\tau } & =\frac{\partial \tau }{\partial t}+v\;\cdot \;\tau -\nabla v^{T}\;\cdot \;\tau -\tau \;\cdot \;\nabla v \end{array}$$

(A12) $$\begin{array}{cc} \lambda \left(T,\dot{\gamma }\right) & =\frac{\eta \left(T,P,\dot{\gamma }\right)}{G} \end{array}$$

with *G* = 6.6 $\times \;10^{5}$ Pa.

### Appendix A.6. Structure VE: Generalized Maxwell Model

(A13)
$$G\left(t\right)=G_{\infty }+\sum_{i=1}^{n}G_{i}\;\exp\left(-\frac{t}{\lambda_{i}}\right)$$

(A14)
$$\lambda_{i}\left(T\right)=\lambda_{i}\left(T_{f}\right)\;\cdot \;a_{T}\left(T\right)$$

(A15)
$$a_{T}\left(T\right)=\left\{\begin{array}{cc} \exp\left(\frac{-A_{1}\left(T-T_{f}\right)}{A_{2}+\left(T-T_{f}\right)}\right), & \mathrm{if}\;T\geq T^{*}\\ \exp\left(\frac{\Delta H_{t}}{R}\left(\frac{1}{T}-\frac{1}{T_{0}}\right)\right), & \mathrm{if}\;T<T^{*} \end{array}\right.$$

**Table A5:** Parameter values for Generalized Maxwell Model.

Parameter	Value	Unit
$A_{1}$	41.9	-
$A_{2}$	51.6	K
$T_{f}$	368.15	K
$T^{*}$	368.15	K
$T_{0}$	368.15	K
$\frac{\Delta H_{t}}{R}$	36.4 $\times \;10^{3}$	K
$G_{\infty }$	1	Pa

**Table A6:** Prony series parameter values for Generalized Maxwell Model.

No.	*G* [Pa]	λ [s]
1	3.98 $\times \;10^{8}$	0.02
2	1 $\times \;10^{9}$	1
3	5.62 $\times \;10^{8}$	10
4	6.31 $\times \;10^{7}$	200
5	4.47 $\times \;10^{7}$	1 $\times \;10^{5}$
6	1.26 $\times \;10^{6}$	5 $\times \;10^{6}$
7	5.01 $\times \;10^{5}$	1 $\times \;10^{8}$
8	5.94 $\times \;10^{5}$	2.15 $\times \;10^{9}$
9	3.95 $\times \;10^{5}$	5 $\times \;10^{10}$
10	1.18 $\times \;10^{5}$	1 $\times \;10^{12}$

### Appendix A.7. Other Mechanical Properties

**Table A7:** Mechanical properties.

Parameter	Value	Unit
Elastic modulus	2.872	GPa
Poisson’s Ratio	0.388	-
Coefficient of Thermal Expansion	5.91 $\times \;10^{-5}$	1/K
